# Participation in a pre-registration student interprofessional education (IPE) society: influence on subsequent professional practice

**DOI:** 10.3389/fmed.2024.1497799

**Published:** 2025-01-28

**Authors:** Christine Hirsch, Emily Audet, Ekrahh Dawood, Freya Beardmore, Nafeesa Hussain, Wing Chi Wong, Robert J. Barry, Sharon Buckley

**Affiliations:** ^1^College of Medical and Health Sciences, University of Birmingham, Birmingham, United Kingdom; ^2^Royal Wolverhampton NHS Trust, Wolverhampton, United Kingdom

**Keywords:** interprofessional education (IPE), pre-registration, student societies, influence on practice, professional practice, health professions, student organizations

## Abstract

**Background:**

Student interprofessional education (IPE) societies or organizations are popular ways to support pre-registration health professions students to develop the understanding and skills needed for collaborative working. Our experience with the University of Birmingham Knowledge and Skills Exchange (KASE) is that, whilst such societies can be excellent vehicles for IPE, sustaining them can be challenging; and that consistent faculty support, adequate resource and a focus for society activities are needed for them to flourish. Whilst the longer term impact of pre-registration IPE has been demonstrated, less is known about the influence of student IPE society membership on participants’ subsequent professional practice. To inform institutional decisions about establishing and maintaining a student IPE society, we have investigated the perceptions of early career health professionals who were KASE members during their pre-registration training.

**Methods:**

KASE alumni working as early career health professionals were invited to participate in the study. Their perceptions of the influence of KASE on their transition to practice and experience as early career health professionals were explored through online semi-structured interviews and interview transcripts analyzed thematically. Resulting themes were reviewed for relevance to the University of Birmingham (UK) IPE Framework, which uses the competency domains of the Canadian Interprofessional Health Collaborative Competency Framework as the required learning outcomes for IPE at Birmingham.

**Results:**

Eight interviews with former KASE members were conducted between November 2022 and March 2023. Interviewees had between 2 and 5 years of experience in their professional role. Six themes relating to the influence of KASE on their subsequent professional practice were identified: interprofessional communication, teamworking, patient-centered care, leadership and organizational skills, confidence and resilience. Three of these themes related to required IPE learning outcomes. Two further themes: time to build relationships; and informality and autonomy, suggested possible reasons for such influence.

**Conclusion:**

Early career health professionals considered that participation in the KASE student IPE society helped their transition into the healthcare work environment and encouraged them to adopt a more collaborative and patient-centered approach. Benefits reported suggest that faculty support for institutional student IPE societies is worthy of consideration.

## Introduction

Student interprofessional education (IPE) societies, known in some contexts as student IPE organizations, are popular ways to support pre-registration health professions students to develop the understanding and skills that they need to work as members of multi-disciplinary teams [(1), Supplementary Appendix 1]. Such societies may have a single focus of activity (2–4), or, like our University of Birmingham Knowledge and Skills Exchange (KASE), engage in a range of activities as the interests of members and resources of the society allow.

Our experience with the University of Birmingham Knowledge and Skills Exchange (KASE) (5) is that, whilst such societies can be excellent vehicles for IPE when working well, sustaining them in a context of rapid student ‘turnover’ can be a challenge; and that consistent faculty support, adequate resource and a focus for activities are needed if they are to grow and develop over time. As such, student IPE societies are not ‘cost neutral’ either for students or staff; and it is important to know whether the commitment that both groups make to their society has the desired effects on subsequent clinical practice. In short, is establishing a student IPE society in a university a good use of time and resources?

Investigations of the long-term impact of pre-registration IPE provide evidence for its positive impact on clinical practice and patient care (6–9). However, whilst some authors (10) have studied the initial impact of participation in an interprofessional student society, few have considered longer term influence on transition to practice and experience as an early career health professional.

The University of Birmingham pre-registration IPE Framework provides for core and optional IPE activities at three levels: raising awareness, knowledge and skills building, and application to practice (11). Required IPE learning outcomes at Birmingham are those of the Canadian Interprofessional Health Collaborative IPE Framework (12). All pre-registration health professions students may participate in the Birmingham Knowledge and Skills Exchange (KASE) as an optional IPE activity. KASE members have participated in team building activities, including healthcare team challenges, student-led workshops and case discussions, a weekend at an outdoor pursuits center and volunteering with local charities and elderly care environments. KASE members also contribute to development of formal IPE through membership of the Birmingham IPE Steering Group; and some have had the opportunity to share their KASE experience through conferences and publications.

Established in 2015, The Birmingham Knowledge and Skills Exchange has existed for almost 10 years and the founding members are now early career health professionals. To identify any long-term effect of participation in a student-led IPE society, we have investigated their perceptions of how KASE has influenced their subsequent experience in clinical practice, focusing particularly on how KASE has influenced their transition to the healthcare work environment, preparedness for their role(s) in the healthcare team and approach to teamworking and patient care. The outcomes of our study will help to inform institutional decisions about whether to set up a student IPE society.

## Materials and methods

KASE alumni who were working as early career health professionals were invited to participate via email, social media or through personal contact. Full information, including a participant information sheet and consent form were emailed to those who expressed an interest in taking part. All participants gave informed consent in advance of the interviews.

Semi-structured interviews were conducted over Zoom at a time convenient to the interviewee. Each lasted approximately 40 minutes and was conducted by two student members of the research team (ED and GW or FB and NF), who were the successors and “near-peers” of the interviewees. Open questions explored interviewee’s perceptions of the impact of KASE on their professional development, their teamwork and approach to patient care (for interview schedule, see Supplementary Appendix 2). All interviewers were trained in interview techniques in advance of data collection and were debriefed by faculty members of the research team after each interview. Interviews were recorded and transcribed in Zoom and the transcription checked for accuracy against the audio recording. Pseudo-anonymized transcripts were stored on a secure server.

Data were analyzed thematically using the Framework Method (13) and Microsoft Excel 2016. The Framework Method is a well-known “Codebook” approach to thematic analysis (14) that is suitable for analysis of semi-structured interview data in projects with multiple researchers who have varying levels of analytical expertise (13). Following transcription (Stage 1) and familiarization (Stage 2), student researchers (ED, FB, NH, WW) undertook initial coding of all transcripts (Stage 3). They then worked with CH and EA to develop and apply an analytical framework in order to ensure consistency of coding (Stages 4 and 5). CH and EA identified and summarized groups of related codes (categories) and prepared a tree diagram of potential themes (Stage 6). All members of the team discussed and agreed the final themes (Stage 7).

Agreed themes were compared to the learning outcomes for each domain of the Birmingham IPE framework and relevance of themes to each domain noted.

## Results

Eight KASE alumni were interviewed between November 2022 and March 2023. Participants were from medicine (1), dentistry (1), nursing (1), pharmacy (3), physiotherapy (1) and clinical psychology (1). All had been KASE members during their pre-registration training and in addition had been KASE committee members for between 6 months and 3 years. At the time of interview, all had worked in interprofessional teams for between 2 and 5 years after leaving university and all had continued to work interprofessionally within their scope of clinical professional specialization.

### Influence of KASE on subsequent professional practice

Through Framework analysis, six themes relating to the effect of participation in KASE on subsequent professional practice were identified. These were: interprofessional communication, team working, patient-centered care, leadership and organizational skills, confidence and resilience. Interviewees reported that these aspects of professional practice were fostered by KASE activities such as team building weekends, educational and social events, volunteering (in both homeless and elderly residential living contexts), and committee work.

#### Interprofessional communication

Interviewees reported that their participation in KASE had helped them to develop their ability to communicate well with other professionals, to the benefit of their subsequent clinical work. This included their ability to listen actively and adapt their language as needed, to understand different perspectives and to explain their own role to others:

“*knowing how to communicate with professionals that aren’t my own.*” 04 Nursing

“*in the team, I’m also more likely to listen [and to ask], “what do you think?” It’s important to listen to other people and KASE taught me that.*” 01 Medicine

“*more confidence to present my role and understand it and explain things that people maybe don’t know about it.*” 03 Pharmacy

These benefits translated into improved ability to undertake handovers with a range of professions and to improved communication with patients:

“*We would have handovers in the morning with the nursing team. So the language used was appropriate for nurses.*… *Then we do a handover specifically with the medical team*… *and it’s the same with the physiotherapists as well. If there’s anything to hand over to them, it would be in their language*” 04 Nursing

“*because I’ve seen the holistic way that MDT were done through the KASE challenge*… *explaining decisions to patients*… *making sure they understood it properly when we’re making a decision on medicine for example.*” 03 Pharmacy

#### Teamworking

Interviewees reported that involvement with KASE assisted their ability to work in a multi-disciplinary team by making them more aware, respectful and appreciative of the roles of others:

“*I’m a better person because of KASE*… *I’m more likely to do the easy stuff. [To] say thank you. Say sorry. Say please*… *And just (be) appreciative of what they do*” 01 Medicine

“[KASE] *made me more appreciative of struggles that the professions go through. I think you tend to think that your profession has it [the] worst*… *But*… *everyone has their own difficulties in their profession*” 04 Nursing

“*the biggest takeaway from my experience with KASE is [the importance of] collaboration and communication within the team and mutual respect.*” 08 Physiotherapy

They were also more aware of the importance of team dynamics, the challenges of team hierarchies and the importance of a holistic, team approach to patient care:

“*having the experience [with KASE] has shifted my perspective of it [the team] being at least a significant hierarchy* … *there is nothing better than having the team dynamic be such that people can speak without feeling judged or they are not afraid*… *of feeling spoken down to*” 08 Physiotherapy

“*[KASE] helps you understand that everyone’s role is contributing something*… *So to take a holistic view of patient care and to try and step back*… *see the bigger picture*” 03 Pharmacy

#### Patient-centered care

These insights into teamworking helped interviewees to be more patient-centered in their approach and to see the patient as an integral member of the team:

“*I remember there’s this one lady*… *[who was] struggling mentally, and I thought, where can I go to help this lady?*… *Because of my knowledge of chaplaincy, through*… *KASE*… *I ended up doing that referral, and it was really helpful*.” 04 Nursing

“*And I think KASE did a lot of that for me*… *I don’t even think about it [the team] as a multidisciplinary team, I just think this person knows the most about that*,… *I’m going to go and speak to them*… *I’m going to go and discuss with them what they think would be best*” 07 Pharmacy

“[the] value of collaboration, and you included patients and family members in that … they are a part of the multidisciplinary team and maybe working with KASE helped me see that earlier than some of my colleagues who are transitioning in” 08 Physiotherapy

#### Leadership and organizational skills

All interviewees reported that participation in KASE had helped to develop their leadership and organizational skills, including time management, delegation and management of meetings:

“*being a (KASE) committee member*… *allowed me to get to know the different professions even more, and also [to] improve my own organizational skills.*” 05 Pharmacy

“*being involved in a society*… *helps with leadership, becoming more assertive and working as a team with healthcare professionals that I did end up working with 2 years later.*” 02 Dentistry

Some interviewees also reported that their positive experience with KASE encouraged them to pursue career development opportunities that required interdisciplinary working and to become an advocate for IPE societies:

“*When that [multidisciplinary project] opportunity came up, I jumped on it.*… *And being able to [say]‘it needed to be interdisciplinary’ right? You’re not going to manage that with one perspective.*” 06 Clinical Psychology

“*It’s hard sometimes to encourage people to do so [join KASE] because people like to stick with their own group. That’s just natural. But I would definitely recommend it. It will help you clinically. You think that you know about people’s professions, but you just don’t.*” 04 Nursing

#### Confidence

KASE enhanced interviewees’ confidence to make the transition to clinical practice, particularly the confidence to speak up, to admit that they did not always have a solution, to approach others and to ask for help.

“*I think it [KASE] improved my confidence*2. 02 Dentistry

“* So I’ll go up to a physiotherapist and a pharmacist and just say ‘hello’, you know, ‘how are you all’, ask my question and not be too scared doing it.*” 01 Medicine

“*KASE*… *gave me confidence*… *not (that) I don’t have any fear. But I’m happy to go and ask anyone the questions I have and I’m happy to walk up to someone and say, ‘I’ve just been looking at this. I’m not sure whether it’s a silly question. But could you talk me through?”’* 07 Pharmacy

“[*KASE] gave me the confidence to tell patients that I don’t have a solution, but I know so and so, or I can speak to so and so colleague.*” 02 Dentistry

Several interviewees explained that greater confidence stemmed from building relationships with other professions during KASE activities and coming to see the “person behind the profession.”

“*But the other side of things also is seeing other professionals as not just their profession, but as humans as well.*” 04 Nursing

“*Because of KASE, I go on to the ward, and*… *I see the person, and then the profession second.” 05 Pharmacy*

#### Resilience

Interviewees recognized that KASE had helped them to develop their resilience; and identified several ways in which this had happened: they appreciated more the importance of asking for help and of having an adaptable approach to changing circumstances; and through their improved understanding of others’ roles, they appreciated that they could help others in their turn:

“*I think as part of (being in) KASE I’m more likely to ask for help*… *So if I had*… *a drug question, I’d approach the pharmacist first*… *I am more likely to ask for help in places that I didn’t know existed, or I knew they existed, but I probably wouldn’t have approached them.*” 01 Medicine

“*So I think KASE it, it’s hard to quantify but it’s more the kind of*… *adaptability*… *I used to work in [Trust X] as [a junior doctor*,… *the MDT team working there was really useful but really easy as well*… *then coming to [Trust Y, it] is very busy*… *but also very understaffed. you kind of need to know who to ask for what*… *the understanding and the ability to change*” 01 Medicine

“*As I left Uni, I was asked to shadow her [the Physician Associate] for a little bit*… *she was just like “Oh, I bet you don’t even know what I do.” I said, “actually, I do. You do this, this, this and this.*”… *And it opened up that dialogue which wouldn’t have been there before.*” 05 Pharmacy

### Enabling features of KASE

Analysis identified two further themes that suggested why KASE fostered the development of more collaborative professional practice: time to build relationships with individuals from other professions and informality and autonomy.

#### Time to build relationships

Interviewees considered that the nature of KASE, in which members worked with each other to plan and carry out activities over weeks, months and (in some cases) years, enabled them to build relationships that led to greater understanding of, and ability to communicate with, other professions:

“*With KASE, it was a couple of years*… *You’re working with everyone in the team for each of the goals that you set*… *creating those connections was more beneficial compared to having say two weeks where there’s no continuity. Just doing that for a couple of weeks doesn’t give you the insight.*” 02 Dentistry

“*And then, we all became friends, you spend a lot of time together. You chat with people and*… *you’ll see the same people every 3 weeks. You get to know them as people.*” 06 Clinical Psychology

“*[On placement] You don’t really spend enough time with them [other healthcare students] to have any kind of real relationship, whereas [in KASE] we were creating relationships with each other.” 07 Pharmacy*

#### Informality and autonomy

Learning informally and socializing in KASE enabled interviewees to ask questions and share their experiences; and being able to decide what activities to pursue and how, allowed them to address the interests and concerns of members:

“*KASE allowed conversation between professionals. It allowed just more organic learning, nothing so structured and just spending time with the professionals.*” 04 Nursing

“*We chose all the topics, we worked together to choose things that we wanted to talk about. Asked the people in KASE what they wanted to learn about. but [with the] emphasis on getting to know them as people*” 07 Pharmacy

“*[With KASE] you are taken out of the academic environment, and you get to know people socially, which benefits professional relationships. The kind of challenges that we did as well, [it] wasn’t just, you know, clinically focused*… *but just general problem solving.*” 03 Pharmacy

### Relevance of themes to interprofessional competencies

The content of three of the themes identified relate to five of the six domains of the Birmingham IPE Framework, which uses the competencies of the Canadian Interprofessional Health Collaborative (CIHC) Framework as its required learning outcomes. These are Interprofessional Communication, Team Working and Patient-Centered Care (Table 1).

**TABLE 1:** Relevance of themes to interprofessional competencies.

IPE framework competency domain	Overview of domain learning outcomes	Relevant study theme (example quote)
Patient-centered care	To seek out, integrate and value, as a partner, the input, and the engagement of the patient/client family/community in designing and implementing care/services.	Patient-centered care “[*the*]*value of collaboration, and you included patients and family members in that*…*they are part of the multi-disciplinary team and maybe working with KASE helped me to see that earlier than some of my colleagues who were transitioning in.*” 08 Physiotherapy
Interprofessional communication	To communicate with different professions in a collaborative, responsive and responsible manner.	Interprofessional Communication “*in the team I’m more likely to listen and [ask] ‘What do you think?’ it’s important to listen to other people and KASE taught me that.*” 01 Medicine
Role clarification	To understand their own role and the roles of those in other professions, and use this knowledge to establish and achieve patient/client/family and community goals.	Interprofessional Communication “*more confidence to present my role and understand it and explain things that people maybe don’t know about.*” 03 Pharmacy
Collaborative working	To understand and apply principles of collaborative practice. This domain supports shared decision-making and leadership but also implies continued individual accountability as defined within one’s professional scope of practice.	Teamworking “*the biggest takeaway from my experience with KASE is [the importance of] collaboration and communication within the team and mutual respect.*” 08 Physiotherapy
Team functioning	To understand the principles of teamwork dynamics and group/team processes to enable effective interprofessional collaboration.	Teamworking “*having the experience [with KASE] has shifted my perspective of it [the team] being at least a significant hierarchy* … *there is nothing better than having the team dynamic be such that people can speak without feeling judged or they are not afraid*… *of feeling spoken down to” 08 Physiotherapy*

The required learning outcomes of the Birmingham IPE Framework are those of the Canadian Interprofessional Health Collaborative (CIHC). Domains and descriptions of CIHC competencies are shown, together with related themes and illustrative quotes from this study. Our themes did not relate to the CIHC Domain of Interprofessional Conflict Resolution.

## Discussion

We investigated former members’ perceptions of the impact of their participation in the Birmingham Knowledge and Skills Exchange (KASE) on their subsequent experience in clinical practice, including their transition to the healthcare work environment, preparedness for their role(s) and their approach to teamworking and patient care.

Our findings suggest that participation in KASE can lead to a range of perceived benefits to subsequent practice, including enhanced interprofessional communication, teamworking, patient-centered care, leadership and organizational skills, confidence and resilience. The content of the first three themes relate to required learning outcomes for Birmingham IPE. Given that outcomes for Birmingham IPE are the competencies of the Canadian Interprofessional Health Collaborative Framework, our findings may be of interest to others who use the Canadian framework or others with similar competencies.

Our findings align with those of the Inter Health Professionals Alliance at Virginia Commonwealth University (10) which found that an interprofessional healthcare student-led initiative, involving participation in monthly community outreach projects and hosting student-led campus sessions supported students’ knowledge and skills development, interprofessional networking and professional competence; and with the findings of Fleming and colleagues, who reported benefits to attitudes toward collaborative practice and team working from interprofessional healthcare team challenges (15).

Skills development through participation in uni-professional student societies has also been reported. Zeeman and colleagues (16) mapped skills acquired through participation in three pharmacy student societies to required core competencies and found that such societies offered opportunities to develop up to two thirds of those required for pharmacy, including skills of collaboration. Student IPE societies such as KASE, however, provide added value compared to uni-professional student societies, in that they foster interaction between the different professions who will form multi-disciplinary teams of the future and, as noted by one of our KASE respondents, help students to see the importance of collaboration earlier than their colleagues who had not had the same experience.

The benefits of KASE participation seem to accrue in part from the fact that students were members of KASE over extended periods of time and so had the opportunity to build relationships, indeed friendships, with students from other professions. This intuitive finding aligns with those of Meffe et al. (17), who noted that time is needed for the development of relationships of trust and respect between nursing, midwifery and medical students. Similarly, though of shorter duration than KASE, students taking part in the recent All-Ireland interprofessional healthcare team challenge valued the opportunity over 6–8 weeks to develop relationships with students from other healthcare professions (15).

The informality of KASE activities, whether educational or social, also seems to enable benefits to subsequent practice by encouraging students to share their questions and concerns, characteristics which may relate to theories of active and social learning (18) and the applicability of the contact hypothesis to interprofessional contexts (19). Furthermore Mink et al. (6) reported that students on an interprofessional training ward considered that informal interactions during shared breaks improved interprofessional collaboration and socialization; whilst Meffe et al. (17) discuss the value of informal time during their interprofessional education pilot program in maternity care suggest that these contacts within social groups reduce intergroup prejudice.

The opportunity to direct their own society was valued by interviewees and may have contributed to realization of the reported benefits to clinical practice. The fact that IPE societies such as KASE are student-led, with members undertaking much of the work of event organization and delivery, can be seen as an advantage for overstretched faculty. However, our study suggests that some of the activities that were most valuable, such as the healthcare team challenges and volunteering in local elderly care facilities, were often those that required faculty support to be successful. In this context, it is worth noting that other extra-curricular healthcare team challenges that have reported benefits (15, 20, 21) also involved extensive faculty support.

Undertaking this study has led us to reflect on the term ‘student-led’ and to a greater appreciation that, as Nagel and colleagues noted (22), this is often used as an umbrella term that can encompass a range of contexts: from initiatives that are informed by student needs and preferences but organized by faculty, to those that are student-led, both in terms of content and organization. As a multi-activity society, the position of KASE on this continuum varies, depending on how much faculty support is required for a particular event. In establishing a society, institutions may wish to consider where on this continuum their society will lie, bearing in mind that the ability of a student-led society to be self-sustaining will depend on the focus of its activity or activities. Our view is that some provision for faculty oversight and support should be part of any plans to establish a society.

Benefits accruing from the greater approachability of near-peer tutors have been widely reported in health professions education, including in interprofessional settings (23). In our study, we employed current KASE committee members as near-peer interviewers to help encourage open responses from interviewees. This approach had the added advantage that the student members of the research team were able to gain valuable skills of qualitative research and to learn first-hand about the potential benefits of their commitment to KASE. Again however, faculty support is needed for the benefits of this approach to be realized.

Whilst our interviewees were well placed to comment on the influence of KASE on their subsequent professional practice, our study has several limitations. Some professions may find participation in a student IPE society more beneficial than others and our interviewees may have been more favorably disposed toward KASE than a different professional mix. As volunteers and former KASE committee members, their experience may not fully reflect that of students who took a less active role; and whilst interview questions related to the influence of KASE *per se*, their perceptions may have been influenced by participation in other IPE activities or student societies. Despite our best efforts otherwise, our analysis may have been influenced by our position as health professions educators with an interest in interprofessional education; and whilst our study has identified benefits for subsequent professional practice, we can only infer relevance to rather than achievement of required IPE learning outcomes.

Within the context of these limitations, our study suggests that participation in a student IPE society can benefit subsequent professional practice and signals enabling features of these societies that may be difficult to replicate in other curricular settings. Our findings resonate with our anecdotal experience over the years and suggest that working with students to establish and sustain a student IPE society is a beneficial use of faculty time and resource.

## Conclusion

Early career health professionals considered that participation in the KASE student IPE society helped their transition into the healthcare work environment and encouraged them to adopt a more collaborative and patient-centered approach. Benefits reported suggest that faculty support for institutional student IPE societies is worthy of consideration.

## Data Availability

The datasets presented in this article are not readily available because no permission was gained from participants to share raw anonymized interview data. Requests to access the datasets should be directed to CH, c.a.hirsch@bham.ac.uk.

## References

[1] VanderWielen LM Enurah AS Osburn IF LaCoe KN Vanderbilt AA. The development of student-led interprofessional education and collaboration. *J Interprofess Care.* (2013) 27:422–3. 10.3109/13561820.2013.790882 23679671

[2] Goodier R Uppal S Ashcroft H. Making international links to further interprofessional learning: A student-led initiative for the homeless population. *J Interprofess Care.* (2015) 29:265–7. 10.3109/13561820.2014.944258 25078466

[3] Adams EJ Schroth S Kaundinya T. Student-driven disability advocacy and education within the health professions: Pilot survey results from a single-day virtual conference. *J Commun Healthc.* (2023) 16:255–9. 10.1080/17538068.2023.2208836 37140055

[4] Zeien J Vieira J Hanna J Surendra L Stenzel J Ramirez A Mpox case reports in an urban homeless population and a proof of concept for a street-based mobile mpox vaccination clinic. *J Prim Care Commun Health.* (2023) 14:21501319231169991. 10.1177/2150131169991 PMC10192806 37191007

[5] Audet E Hirsch C Vickneswaran K Ayub M Arain M Norton T Building and sustaining student leadership in IPE: Experience with the knowledge and skills exchange. In: Forman D Jones M Thistlethwaite J editors. *Sustainability and Interprofessional Collaboration: Ensuring Leadership Resilience in Collaborative Health Care.* London: Palgrave Macmillan (2020). p. 251–69. 10.1007/978-3-030-40281-5_14

[6] Mink J Zurek B Götsch B Mihaljevic AL Mitzkat A Trierweiler-Hauke B How do former medical and nursing undergraduates describe their learning on an interprofessional training Ward 12–18 months later?–A retrospective qualitative analysis. *BMC Med Educ.* (2023) 23:275. 10.1186/s12909-023-04212-5 37085857 PMC10122365

[7] Darlow B Brown M McKinlay E Gray L Purdie G Pullon S. Longitudinal impact of preregistration interprofessional education on the attitudes and skills of health professionals during their early careers: A non-randomised trial with 4-year outcomes. *BMJ Open* (2022) 12:e060066. 10.1136/bmjopen-2021-060066 PMC9305815 35858731

[8] Macauley K Skov H Lehtonen K Shulman B. Perceptions of an international interprofessional education experience: Findings from students based in Europe and North America. *J Interprofess Care.* (2016) 30:606–14. 10.1080/13561820.2016.1189888 27341070

[9] McNaughton S. The long-term impact of undergraduate interprofessional education on graduate interprofessional practice: A scoping review. *J Interprof Care.* (2018) 32:426–35. 10.1080/13561820.2017.1417239 29271675

[10] VanderWielen LM Do EK Diallo HI LaCoe KN Nguyen NL Parikh SA Interprofessional collaboration led by health professional students: A case study of the inter health professional alliance at Virginia Commonwealth University. *J Res Interprofess Pract Educ.* (2014) 3:1–13. 10.22230/jripe.2014v3n3a132

[11] University of Birmingham Framework for Interprofessional Education. (2023).

[12] Canadian Interprofessional Health Collaborative. *A National Interprofessional Competency Framework.* (2010). Available online at: https://phabc.org/wp-content/uploads/2015/07/CIHC-National-Interprofessional-Competency-Framework.pdf (accessed September 03, 24).

[13] Gale NK Heath G Cameron E Rashid S Redwood S. Using the framework method for the analysis of qualitative data in multi-disciplinary health research. *BMC Med Res Methodol.* (2013) 13:117. 10.1186/1471-2288-13-117 24047204 PMC3848812

[14] Braun V Clarke V. Toward good practice in thematic analysis: Avoiding common problems and be(com)ing a knowing researcher. *Int J Transgender Health.* (2023) 24:1–6. 10.1080/26895269.2022.2129597 PMC9879167 36713144

[15] Fleming A Mongan O McCarthy M O’Donnell M Lydon A McGowan E Development of a student intervarsity interprofessional healthcare team challenge to promote collaboration and teamwork. *J Interprofess Educ Pract.* (2024) 36:100708. 10.1016/j.xjep.2024.100708

[16] Zeeman JM Bush AA Cox WC Buhlinger K McLaughlin KE. Identifying and mapping skill development opportunities through pharmacy student organization involvement. *Am J Pharm Educ.* (2019) 83:492–500. 10.5688/ajpe6950 PMC6581355 31223160

[17] Meffe F Moravac C Espin S. An interprofessional education pilot program in maternity care: Findings from an exploratory case study of undergraduate students. *J Interprofess Care.* (2012) 26:183–8. 10.3109/13561820.2011.645089 22251306

[18] Khalili H Orchard C. The effects of an IPS-based IPE program on interprofessional socialization and dual identity development. *J Interprofess Care.* (2020): 10.1080/13561820.2019.1709427 [Epub ahead of print]. 32019374

[19] Michalec B Giordano C Dallas S Arenson C. A longitudinal mixed-methods study of IPE students’ perceptions of health profession groups: Revisiting the Contact Hypothesis. *J Interprofess Educ Pract.* (2017) 6:71–9. 10.1016/j.xjep.2016.12.008

[20] Ting D Sutherland A Donnelly C. Students’ experience of the Health Care Team Challenge™: Long-term case competition can improve students’ competence in interprofessional collaboration. *Can Med Educ J.* (2013) 4:e49. 10.36834/cmej.36674 PMC4563603 26451214

[21] Boyce RA Moran MC Nissen LM Chenery HJ Brooks PM. Interprofessional education in health sciences: The University of Queensland health care team challenge. *Med J Austr.* (2009) 190:433–6. 10.5694/j.1326-5377.2009.tb02492.x 19374616

[22] Nagel DA Naccarato TT Philip MT Ploszay VK Winkler J Sanchez-Ramirez D Understanding student-run health initiatives in the context of community-based services: A concept analysis and proposed definitions. *J Prim Care Commun Health.* (2022) 13:21501319221126293. 10.1177/21501319221126293 36164929 PMC9520185

[23] Young M Wilkinson T. Near-peer interprofessional simulation training in an undergraduate setting. *BMJ Simul Technol Enhanc Learn.* (2019) 5:111. 10.1136/bmjstel-2017-000266 35519835 PMC8936990

