# Supplementary material for: Participation in a pre-registration student interprofessional education (IPE) society: influence on subsequent professional practice
Source: Front Med (Lausanne). 2025 Jan 28;11:1497799. doi: 10.3389/fmed.2024.1497799 (PMC11811773; doi:10.3389/fmed.2024.1497799)
Supplement: Supplementary file 1 [file Data_Sheet_1.pdf]

Appendix 1

Examples of student interprofessional education societies

IPE Student Healthcare Organisation	Weblinks [Accessed 21.12.2024]
London Interprofessional Healthcare students association (LIHSA), Ontario, Canada.	https://www.facebook.com/LondonIHSA/
Robert Gordon University (RGU) student IPE society,UK.	https://www.rgu.ac.uk/rgview/student-experience/3309-getting-involved-in-interprofessional-education
The Interprofessional Health Student Organizations (IPSO), United States.	https://www.marshall.edu/interprofessional-education/student-organization/
The World Health Students' Alliance (WHSA).	https://ifmsa.org/world-health-students-alliance/
University of Toronto Interprofessional Healthcare Students' Association (IPHSA), Canada.	https://ipe.utoronto.ca/interprofessional-healthcare-students-association-iphsa https://iphsaipe.weebly.com/
University of Birmingham student IPE society, UK.	https://www.instagram.com/uob_ipesoc/
University of Cardiff IPE student society, UK.	https://www.facebook.com/CUIPESociety/
University of East Anglia (UEA) IPE student society, UK.	https://www.facebook.com/ueaipesoc/?locale=en_GB
