# Supplementary material for: Participation in a pre-registration student interprofessional education (IPE) society: influence on subsequent professional practice
Source: Front Med (Lausanne). 2025 Jan 28;11:1497799. doi: 10.3389/fmed.2024.1497799 (PMC11811773; doi:10.3389/fmed.2024.1497799)
Supplement: Supplementary file 2 [file Data_Sheet_2.pdf]

Appendix 2

Interview Questions Framework

How has participation in a student-led interprofessional education IPE Society (Knowledge and Skills Exchange, KASE) influenced transition to practice and experience of early career health professionals?

Thank you for giving your time to take part in this interview. We expect it to take around 40 minutes.

Just to check that you have had the opportunity to read the participant information leaflet. Do you have any questions? Just to confirm you agree to this interview being recorded as indicated in the information leaflet, and that you have understood that anonymised quotes from the transcripts may be used in the researcher's narrative report.

You have the right to withdraw at any point from the study, or the option to decline answering any questions today. You have the opportunity to withdraw your interview contribution, up until two weeks from today and you would have to notify Dr Audet or Dr Hirsch on the email address indicated on the information sheet. We have your signed consent form. Are you ready to start?

We will start with a few questions about your academic studies, then move onto some questions more specifically about KASE.

1. What healthcare course did you study?
2. Did you go straight from school into your professional undergraduate programme, or did you enter as a graduate?

   What did you do in between? [PROMPT: Which degree/ programme/ experience]

3. Have you had any breaks in the usual progression through the programme e.g. intercalation/ elective experience e.g teaching/ masters.
4. What year did you graduate?
5. Did you have any healthcare experience prior to starting your healthcare course at UoB?
6. Tell us about your roles and responsibilities now. [PROMPT: Teaching fellowship/ management roles/MSc or further study]

Moving on to questions about KASE.

1. What role/roles did you have in KASE?
2. How long were involved with KASE?
3. How did you initially hear about KASE?
4. What attracted you to attending KASE events?
5. Why did you join the KASE committee?
6. What do you remember most about KASE and the opportunities that it gave you?

We now have some questions which ask you to reflect on the impact of your experience in KASE on yourself, teamworking and patient care. We will start with the impact of your experience with KASE on yourself.

Can you please tell me how you feel that your experience with KASE has influenced your development as a healthcare professional, perhaps provide some examples.

Follow up questions where these areas are not covered in the initial response or further detail required

a) Following graduation and training, how well prepared did you feel for working in your clinical role? [PROMPT - what makes you say that? Can you elaborate further?]
b) How do you think that your experience with KASE has contributed to the person/professional that you are today?
c) How do you feel that working with KASE specifically prepared you for clinical work? [PROMPT Can you give an example? PROMPT In any particular way?]
d) What particular skills did you develop from KASE that you have found useful to your career?
e) How do you think the experience and skills developed with KASE differed from those developed from IPE within your healthcare course curricula?
f) How do you feel your experience with KASE impacted your career opportunities?
g) How has your experience with KASE impacted your career choices?
h) How valuable do you think your experience with KASE was to your professional/personal development? [PROMPT, can you give any specific examples?]
i) What would you say about joining an IPE student led society to current healthcare students? [PROMPT Would you recommend joining an IPE-society to current healthcare students? Please explain your answer. What makes you say that?]

Thank you, the next questions are around your reflection on the impact of your KASE-experience on teamworking.

1. Please tell me how you feel that your experience with KASE has influenced the way that you work a as member of the clinical multidisciplinary team. (PROMPT Are there any specific examples?]

Follow up questions where these areas are not covered in the initial response or further detail required.

a) Can you tell me a bit about the environments that you have worked in since graduating and where you are working now? [PROMPT: Has, and is, your work been within an interprofessional environment?]
b) What is your current role, and is this within an interprofessional team?
c) How did your experience with KASE specifically prepare you for clinical teamworking?
d) How did KASE give you teamworking experiences and skills that differed from timetabled IPE delivered in your healthcare course? [PROMPT Please explain your answer.]

The next three questions are about interactions with different teams.

2. How do you think that your experience within KASE has influenced or changed the way that you interact with other healthcare *professionals*?

3. How do you think that your experience within KASE has influenced or changed the way that you interact with other healthcare students?

4. How do you think that your experience within KASE has influenced or changed the way that you interact with non-clinical members of the wider team for example ward clerks, secretaries, other administrative staff, managers?

Carrying on with some general questions about team working.

5. How has your experience with KASE affected the way that you communicate within a team? [PROMPT this may be in your current role or previous roles that you have had]

6. Do you feel able to speak up in a team?

7. Have you sought any interprofessional roles, such as further IPE training or collaborative projects since graduating?

8. Have you had any leadership roles (or roles that you consider to be leadership roles?) since graduating? [FOLLOW UP: What are these?]

9. How do you feel that your experience with KASE helped prepare you for leadership roles?

10. What opportunities have you had to form networks through KASE that may be useful throughout your career (either now or your feel you might use in the future?)

And moving on to some questions about impact of your KASE experience on your relations or working with patient, client or service users.

1. Please tell me how you feel that your experience with KASE has influenced the way that you work with patients, clients and service users. [PROMPT: Are there any specific examples?]

Follow up questions where these areas are not covered in the initial response or further detail required.

a) How did your time with KASE specifically prepare you for providing patient care? [FOLLOW UP: Do you feel that you developed any skills with KASE that have led to improved patient care?
b) How has your experience with KASE has influenced or changed the way that you interact with patients?
c) How has KASE prepared you for working in different work environments and different healthcare situations?

And two final questions.

1. How has your experience of interprofessional working in practice, matched your expectations? Without naming individuals or specific places of work can you give any examples? [PROMPT: This may be expectations as an undergraduate or from previous experience.]

2. Is there anything else that you would like to add that we have not discussed in relation to the impact of your experience of being part of KASE since starting clinical work?

Thank you for your time and input. Just to confirm that if you decide that you wish to retract your interview from the study, you must contact us within two weeks and your transcript will be removed from the study.

If you would like to, we can send a copy of the transcript for your comments on the transcript before we start analysis. Would you like to do this? YES/NO

Would you like to receive a copy of the study outcomes once the study is completed? (This would be sent to the email contact that you have given)

Thank. *Conclude the interview and stop recording.*
